# Dynamically loading IFC models on a web browser based on spatial semantic partitioning

**DOI:** 10.1186/s42492-019-0011-z

**Published:** 2019-06-03

**Authors:** Hong-Lei Lu, Jia-Xing Wu, Yu-Shen Liu, Wan-Qi Wang

**Affiliations:** 1 School of Software, Tsinghua University, Beijing, 100084 China; 2 Beijing National Research Center for Information Science and Technology (BNRist), Beijing, 100084 China; 3 Institute of Computing Technologies, China Academy of Railway Sciences, Beijing, 100081 China

**Keywords:** Building information modelling, Industry foundation classes, IFC models, Dynamically loading online

## Abstract

Industry foundation classes (IFC) is an open and neutral data format specification for building information modeling (BIM) that plays a crucial role in facilitating interoperability. With increases in web-based BIM applications, there is an urgent need for fast loading large IFC models on a web browser. However, the task of fully loading large IFC models typically consumes a large amount of memory of a web browser or even crashes the browser, and this significantly limits further BIM applications. In order to address the issue, a method is proposed for dynamically loading IFC models based on spatial semantic partitioning (SSP). First, the spatial semantic structure of an input IFC model is partitioned via the extraction of story information and establishing a component space index table on the server. Subsequently, based on user interaction, only the model data that a user is interested in is transmitted, loaded, and displayed on the client. The presented method is implemented via Web Graphics Library, and this enables large IFC models to be fast loaded on the web browser without requiring any plug-ins. When compared with conventional methods that load all IFC model data for display purposes, the proposed method significantly reduces memory consumption in a web browser, thereby allowing the loading of large IFC models. When compared with the existing method of spatial partitioning for 3D data, the proposed SSP entirely uses semantic information in the IFC file itself, and thereby provides a better interactive experience for users.

## Introduction

During the last decade, building information modelling (BIM) received significant attention in the domain of Architecture, Engineering, and Construction (AEC) [1]. Additionally, BIM also plays an increasingly important role in smart buildings and smart cities. When compared with CAD, BIM contains geometric and rich semantic information on building models and their relationships to support lifecycle data sharing. Specifically, industry foundation classes (IFC) is an open and neutral data format specification for BIM [2, 3] that describes building and construction industry data and facilitates interoperability between BIM applications. IFC files can be imported or exported through most market-leading BIM software [4]. Recently, studies developed various IFC-based approaches and applications (e.g., refs. [4–9]).

With increases in web-based BIM applications, there is an urgent need for fast loading large IFC models on a web browser [10]. For example, several construction projects require various participants to share and access BIM data through the web where it corresponds to the basic requirement for fast loading IFC files into the web browser and displaying them in real time. There exist a few web-based platforms to manage and display IFC models [11–13] such as the well-known BIMserver [11]. However, the task of loading large IFC models typically consumes a large part of the memory of web browser or even crashes the browser, and this significantly limits further BIM applications. It is still challenging to fast load large IFC models to satisfy specific BIM applications.

Given the memory limitation on the web browser, it is not appropriate to fully load large IFC models [14]. In a few BIM applications, project participants first browse the appearance of BIM model and subsequently interactively select a few local interest parts of the model. Therefore, it is important to set a suitable loading strategy that satisfies these types of applications.

In the study, we present a method for dynamically loading IFC models based on spatial semantic partitioning (SSP). First, the SSP of an input IFC model is pre-computed on the server and is subsequently passed to the client. Next, only the external components of the IFC model are extracted from the nodes of SSP that are initially loaded for displaying the appearance of the IFC model. The spatial partitioning of the model’s stories and establishment of the space index table of components makes it possible to ensure that only the components that are related to the user’s interactive selection are transmitted, loaded, and displayed on the client. The presented method is implemented via Web Graphics Library (WebGL), and this enables large IFC models to be fast loaded on the web browser without requiring any plug-ins. When compared with the conventional methods that load all IFC model data for display purposes, the proposed method significantly reduces the memory consumption in a web browser. This allows the loading of large IFC models and provides a better interactive experience for users.

### Related work

WebGL is a cross-platform web standard JavaScript API for rendering 3D graphics within any compatible web browser without requiring any plug-ins and is widely supported in modern browsers. Recently, a few platforms, such as 3drepo.io [15] and webVis/Instant3DHub [16], began to provide support for commercial web-based 3D rendering. Various efforts focused on transmission format to stream 3D models, such as Shape Resource Container (SRC) [17] and GL Transmission Format (glTF) [18]. However, extant studies do not focus on dynamically loading, and this makes it difficult to load large IFC files on the web browser. Recently, a few studies also examined dynamically loading of 3D data [19–24]. For example, Shen et al. [19] constructed a game scene by using the geometric data of the BIM model. Chen et al. [20] dynamically animated game data via WebSocket. Lavoue et al. [22] implemented a progressive compression algorithm based on user operations on a web browser. Wang et al. [23] proposed a fast dynamic transmission method for 3D cloud data. Li et al. [24] presented a system for the progressive compression and transmission of a 3D model with WebGL. However, the aforementioned transmission methods generally optimize transmission speed via the optimization of data structure and algorithm, and thereby ignores spatial information.

In order to extract spatial information, spatial partitioning is a commonly used method. A few methods were developed to extract the external components based on the bounding box [13]. Scully et al. [14] introduced a spatial partitioning strategy for dynamically loading 3D meshes with the aims of overcoming memory limitations for small mobile devices and those imposed by browsers. However, the aforementioned approaches only focus on the geometric information of 3D objects and ignore the semantic information present in IFC models. A few studies also discuss spatial partitioning for 3D data of shape or scene by combining specific semantic information. For example, Held et al. [25] proposed a method that incorporates spatial, temporal, and semantic cues in a coherent probabilistic framework for spatial partitioning. Babacan et al. [26] proposed a semantic segmentation method for indoor point clouds via a convolutional neural network. However, the aforementioned semantic partitioning methods mainly focused on extracting and generating semantic information for 3D data of shapes or scenes in their applications, and this is not appropriate for IFC models. Specifically, a large amount of rich semantic information is originally carried by IFC files in addition to their specification, and this includes information involving types of spaces, properties of building components and building functions, and various relationships between building components. Thus, this type of semantic information potentially provides a wealth of a priori knowledge for the SSP of IFC models, and this corresponds to the major difference between IFC models and other general 3D data.

### Contributions

Based on ref. [14], we present a method for dynamically loading an IFC model on the web, and this is based on SSP. Spatial partitioning is used in ref. [14] and only partitions 3D geometric meshes into a few submeshes without further considering the semantic information carried by the IFC model itself. Conversely, the proposed method partitions an IFC model by simultaneously considering its geometric information and semantic information in the IFC model itself. The main contributions of our study are as follows.

First, a method is presented for dynamically loading IFC models on the web. When compared with the conventional methods that load all IFC model data for displaying, the proposed method significantly reduces memory consumption in a web browser, and this allows the loading of large IFC models.

Secondly, a novel SSP method is presented for the IFC model, and this constitutes the core of the study. When compared with the existing method of spatial partitioning for 3D data, the presented SSP maximizes the use of semantic information carried by the IFC file itself, and this provides a better interactive experience for users.

Finally, the presented method is implemented via WebGL, and this enables large IFC models to be fast loaded on the web browser without requiring any plug-ins.

## Method

With respect to large IFC models, it is challenging to load and display all the model data on the client. In several practical applications, BIM participants typically only focus on the specific parts and components while browsing the IFC model, and thus a possible method is to dynamically load and display a sub-model as opposed to the full model. This is performed by dynamically loading a few building components of the IFC model based on a user’s interactive selection.

In this section, a method for dynamically loading IFC models based on SSP is proposed. The semantic partitioning of the model story and establishment of the component space index table allows only the model data that the user is interested in to be transmitted, loaded, and displayed. The spatial partitioning referred to in the study is mainly based on semantic information of the IFC model, and this includes the internal and external space of the IFC model, different story spaces, and the space generated via model bounding box partitioning.

**Fig. 1:**
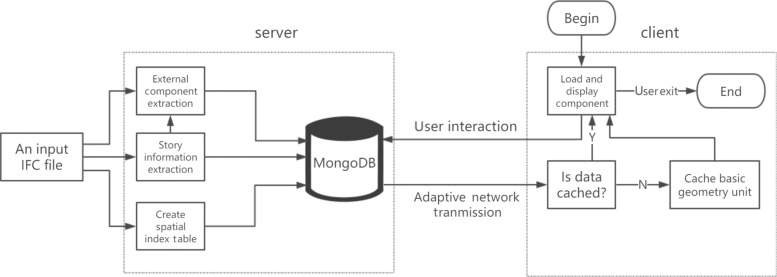
Structure diagram of the proposed method

The structure diagram of the proposed method is shown in Fig. 1. First, the spatial semantic structure of an input IFC model is partitioned via the extraction of story information and establishment of the component space index table on the server. Subsequently, based on the user interaction, we dynamically calculate the list of components and obtain the corresponding components from the server. Next, we use the adaptive network transmission algorithm to transmit basic geometric units to the client, and the basic geometry unit is cached. Finally, the basic geometry unit is loaded and displayed on the client.

### Semantic partitioning of IFC model spatial structure

The partitioning of an IFC model spatial structure mainly consists of the following three operations: external component extraction, extraction of story information, and establishment of spatial index. The operations are all on the server side. We establish the spatial index based on the bounding box of IFC model, and this makes it easy for users to browse specific parts of the IFC model.

### External component extraction based on node classification

When BIM participants browse the IFC model, the external structure is more attractive. When the users explore the model, they focus on the details of the model. Based on the observation, while displaying the IFC model, the external components of the IFC model are preferentially loaded, and the external structure of the IFC model is quickly displayed, thereby effectively improving users’ browsing experience.

**Fig. 2:**
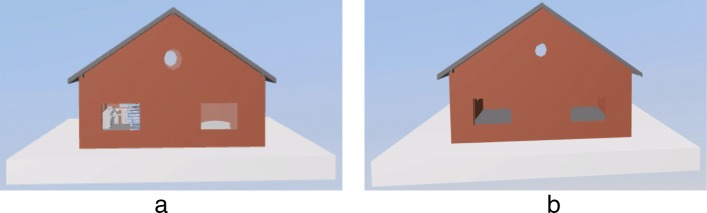
External component extraction based on the bounding box. The external windows and doors are extracted incorrectly. **a** Original model **b** Result based on the bounding box algorithm

Currently, there are a few methods for extracting the external components of the IFC model, and a few methods were developed to extract the external components based on the bounding box [13]. The algorithm first calculates the bounding box of each building component and the bounding box of the whole model. Subsequently, the minimum cover set of each face is calculated via the projection method from six directions. For example, in order to calculate the minimum cover set of the front of the model (assumed to be the *xy* plane), it initially sorts the bounding boxes of all the components of the model along the *z*-axis and calculates the projection area of each component bounding box in the *xy* plane from near to far. Subsequently, if the component does not cover the previously covered area, then it is added into the minimum cover set. Finally, the six faces obtain the cover set and external components. The external component extraction algorithm based on the bounding box omits a few external components in a few cases as shown in Fig. 2.

The main reason for the aforementioned omission of the algorithm is that each component is abstracted into a bounding box to increase the volume of the component, and only the area covered by the projection is considered. As shown in Fig. 2, the bounding-box based extraction algorithm omits the window. This is because the doors and windows are embedded inside the wall while their bounding boxes are contained in the wall’s bounding box. Therefore, they do not appear in the extracted external component. In order to avoid this type of a situation wherein building components are missed, we propose an external component extraction algorithm based on node classification. The pseudo-code of the algorithm is shown in Algorithm 1.

**Fig. 3:**
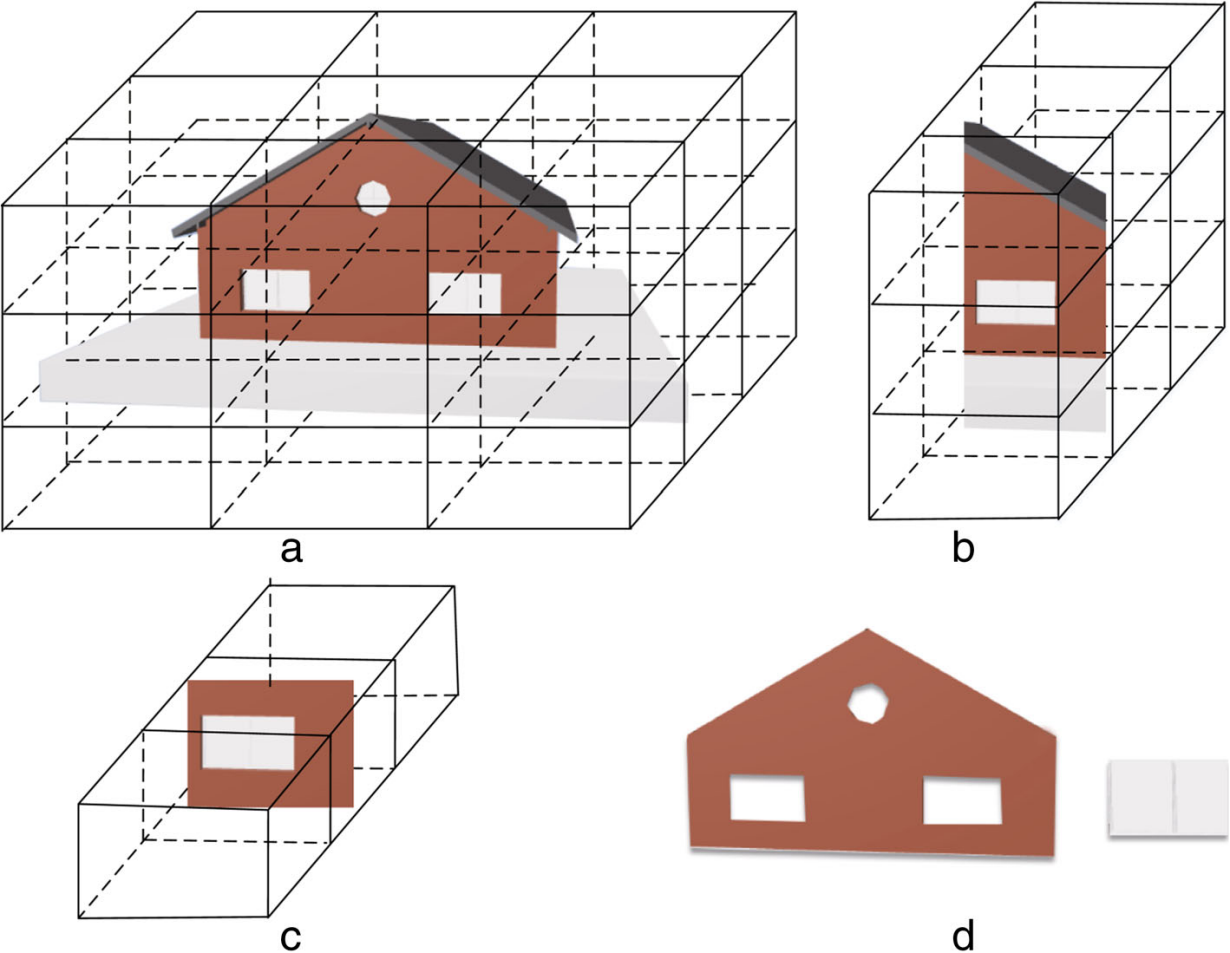
Extracting external components based on node classification. The external doors and windows are extracted accurately. **a** Bounding box division **b** Choose a random column **c** Traversal from near to far **d** Get component


The algorithm initially calculates the bounding boxes of all the components and merges them to form the bounding box of the complete IFC model. Subsequently, the length and width of the bounding box of the model are divided by *t* (the number of SSP) and the bounding box space is divided into *t*^3^ small cube nodes of equal size as shown in Fig. 3a where the whole bounding box of the model is divided into multiple cube nodes of equal size. We then calculate the intersection of each small cube node with all the components in the model. The external part is calculated from one of the six faces (marked as face *P*) of the bounding box. The details of the process are as follows. First, the small cube nodes in the *t* rows and *t* columns on the face *P* are numbered as *P*_11_...*P*_1*t*_...*P*_*t*1_...*P*_*tt*_, and subsequently they are processed followed by a row and column as shown in Fig. 3b and c. With respect to a certain node *P*_*mn*_, it is determined as to whether each small cube node contains any component behind *P*_*mn*_; if so, it indicates that the small cube obscures the component behind *P*_*mn*_. The component contained by the small cube corresponds to the external component in the direction of face *P*. The cube and its subsequent d cubes are marked as shown in Fig. 3d. We then obtain the components contained by the marked cubes and stop searching in this direction. If there is no component, we continue to determine as to whether the next small cube node contains a component. When all small cube nodes *P*_11_...*P*_1*t*_...*P*_*t*1_...*P*_*tt*_ are completely processed, all the external components in the direction followed by *P* are obtained.

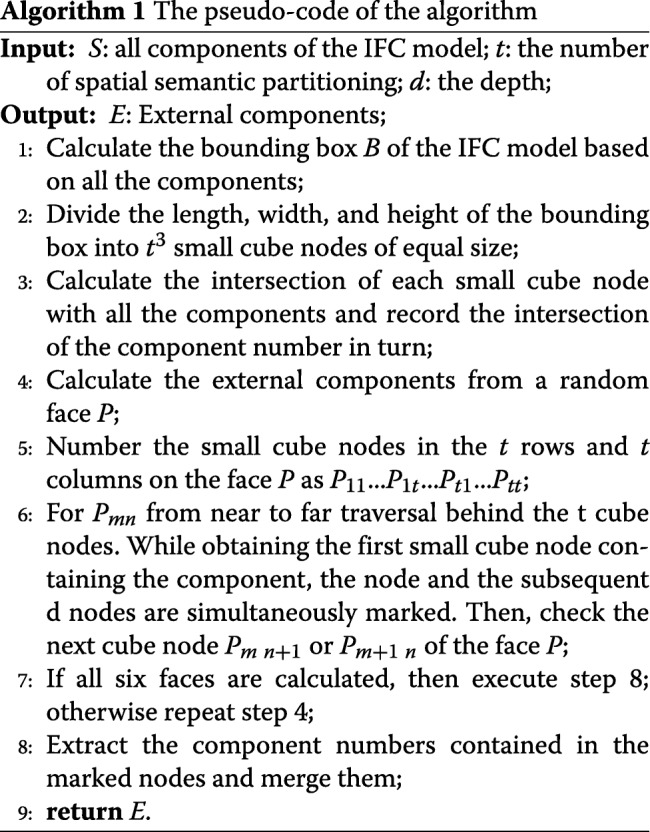


**Fig. 4:**
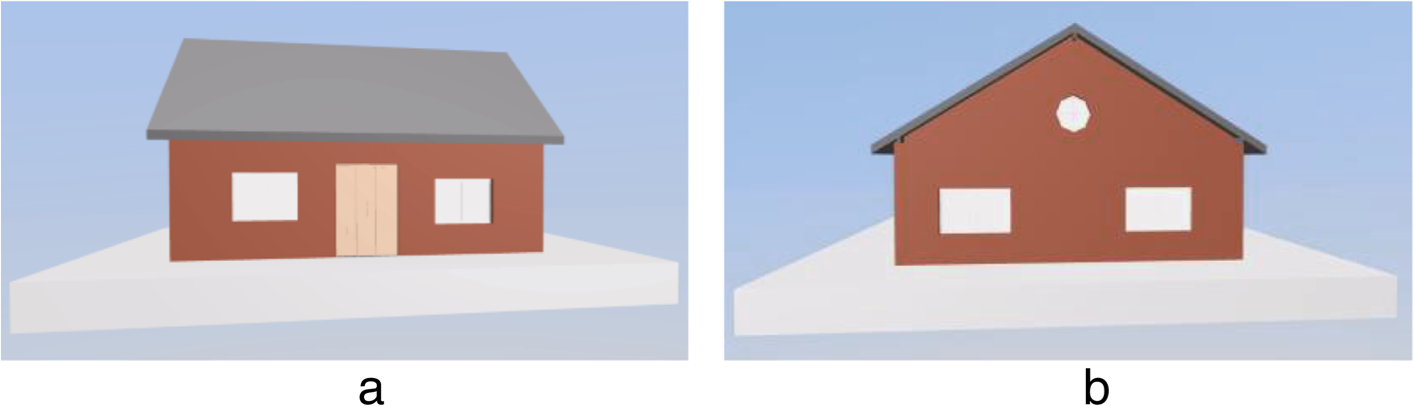
Results after extracting the external components on different faces. All external building components including bases, roofs, walls, and windows are extracted accurately. **a** Front **b** Side

Figure 4 shows the results of the extraction of the two faces of the model. As shown in Fig. 4, both sides of the extraction result contain the correct external windows and doors. However, Fig. 4a and b both contain the bases and roofs. Therefore, after extracting the external components on the six faces, it is necessary to remove duplicate components in the results of the six faces, and only one of the same external components is retained in the final result. Finally, all the indexes of the external components of the obtained model are stored.

The external component extraction algorithm based on node classification considers the depth information although a few members of the bounding box are included. The cube node still intersects the component, and this avoids the omission of external components due to the overlapping of the bounding box.

#### Extraction of story information

The IFC model story information is mainly expressed via the entities of IfcBuilding and IfcBuildingStory that correspond to two types of nodes in the IFC model tree. The IfcBuilding expresses the concept of a virtual architectural spatial structure in the IFC standard, and IfcBuildingStory expresses the concept of a story and a few local spaces on the story. In an IFC model of an actual project, IfcBuildingStory is typically associated with IfcBuilding. An IfcBuilding entity can contain multiple IfcBuildingStroery entities and an IfcBuildingStory entity can also contain other IfcBuildingStory entities that form a tree-like hierarchical structure. The algorithm for story information extraction is shown in Algorithm 2.


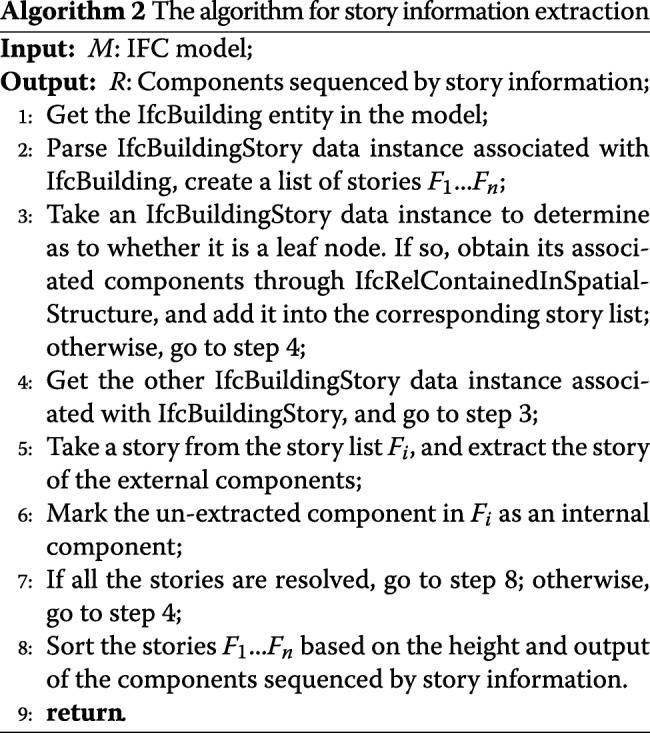


**Fig. 5:**
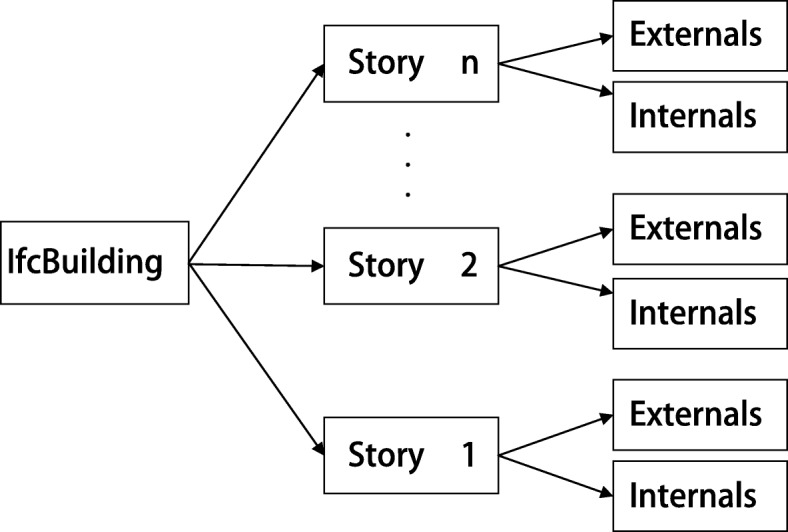
Illustration of the entity IfcBuilding divided by the stories information

The story information is initially extracted via by the IfcBuilding entity of the IFC model that is used as the root node of the depth-first search. We then obtain all the IfcBuildingStory entities contained by IfcBuilding. Subsequently, with respect to each IfcBuildingStory entity, it is determined as to whether the entity is a leaf node. If so, all the components contained in the story entity are obtained by parsing the IfcRelContainedInSpatialStructure (the node contains the inclusion relationship); otherwise, the IfcBuildingStory entity contained in the story is obtained. The process is executed recursively until all story information is resolved. Subsequently, we respectively extract the external components of each story. As shown in Fig. 5, the components included in each story are divided into external components and internal components.

#### Spatial index table

The spatial index table is created to facilitate the users to quickly navigate through the model locally. In order to allow more devices to load and display the model, we preload only the external components of the model or a specific story of the external components for display purposes. The details of the model are not loaded. The users can use the spatial index table when they want to view the components in detail.

**Fig. 6:**
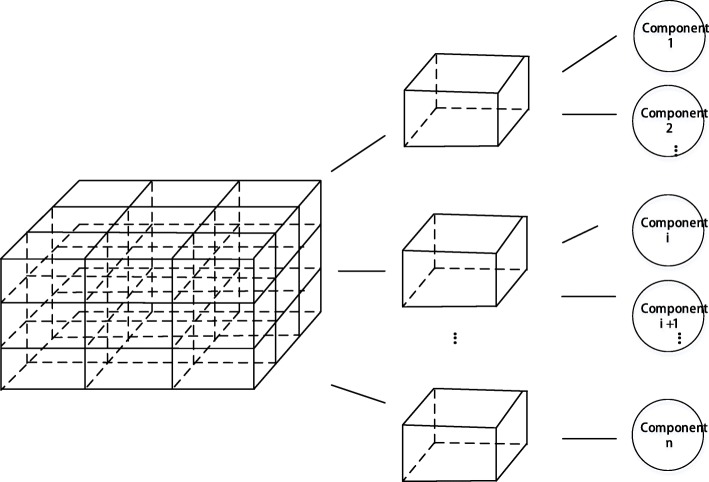
Spatial index table

First, the bounding box of the model is obtained as mentioned in the previous section, and a series of equal cubes are obtained based on the length and width. Subsequently, for each small cube, we determine all components that it intersects with. The cube without a component is removed. After preloading the external components, the small cubes are loaded and displayed. An octree is created in the scene, and the small cubes are added to the octree. A few optimizations are performed to ensure that multiple cube pairs do not repeatedly consume client resources. The size of the small cube is the same, and thus only the geometric data of a cube is stored in the display, and other small cubes are displayed via the coordinate transformation. Finally, when the user enters the model and wants to display a part of the model, only the small cube of the region is selected to obtain the corresponding geometric data of the components from the remote server. Figure 6 shows a schematic diagram of the created component space index table.

### Dynamically loading via adaptive network transmission

The remote server begins to transfer the corresponding model data when the users select a component of the specific spatial structure interactively on the web browser. The data contains several basic geometric units that consume significant bandwidth. Therefore, it takes a long time for the browser to respond, and this affects user experience. Therefore, an adaptive network transmission algorithm is proposed as shown in Algorithm 3. When the user selects the component to be displayed, the model changes to the maximum possible extent in a uniform time period. This decreases the size of data block when the network is not smooth and increases the size of data block when the network is smooth.


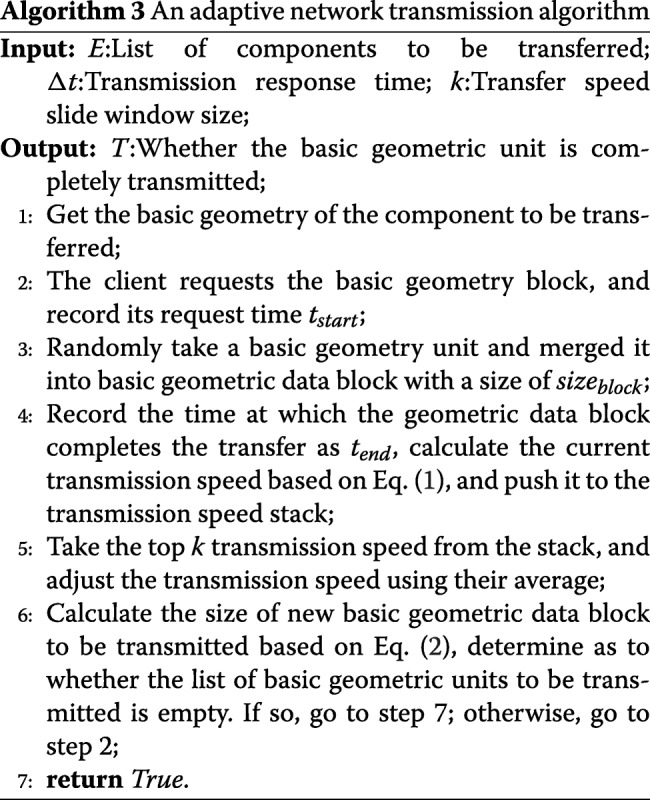


When the user selects a specific component to display, the client first loads the basic information of the components including the basic attributes, basic geometric unit number, and positioning matrix. Subsequently, the basic geometric unit is loaded by the adaptive network transmission algorithm. When all the basic information of the model is transmitted, the client begins to request the geometric data corresponding to the component. First, the client records the requested time *t*_*start*_ at the time of the request and records the current basic geometric data block transfer completion time *t*_*end*_ after the transfer is complete. Based on Eq. (1), we calculate the current basic geometric data block transmission speed and push it to the transmission speed stack. Subsequently, we select the top k transmission speed to smooth. In order for the web page to be updated in the time corresponding to *Δt*, the size of the basic geometric data block to be transmitted next is dynamically adjusted based on Eq. (2), and the aforementioned process is repeated until the transmission is complete. In order to avoid re-downloading the geometric data from the remote server, the basic geometric unit of the model is cached.

1 $$\begin{array}{@{}rcl@{}} speed_{current} &=& \frac{size_{block}}{t_{end} -t_{start}}  \end{array} $$

2 $$\begin{array}{@{}rcl@{}} ceiling_{dynamic} &=& \frac{(\Sigma_{1}^{k} speed)}{k}*\Delta t  \end{array} $$

## Results

**Fig. 7:**
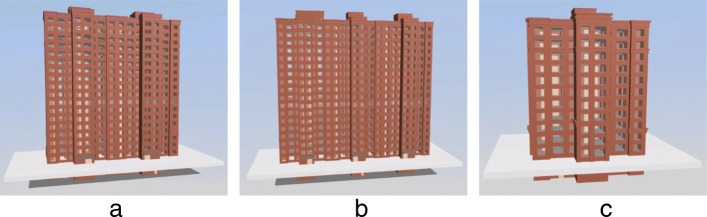
Three test models for loading and displaying. **a** M1 **b** M2 **c** M3

We implemented our algorithm based on WebGL that can run on most mainstream web browsers that support WebGL (including Firefox, Chrome, IE11). Specially, we run our experiments on Firefox browser (64bit) on Windows 7 Operating System (64bit) with CPU i5-3470 and 16 GB memory. In order to analyze the effect of the dynamically loading method based on the SSP of IFC models, we select three large models to test and analyze the resource occupation, loading speed, display effect, and operation fluency. The basic information of the three test models is shown in Table 1, and the corresponding display is shown in Fig. 7.

**Table 1:** Basic information for the three test industry foundation classes models

Model name	Figure	Size(MB)	Number of components
M1	a	157	9105
M2	b	278	13379
M3	c	171	7533

### Analysis of resource occupation

**Fig. 8:**
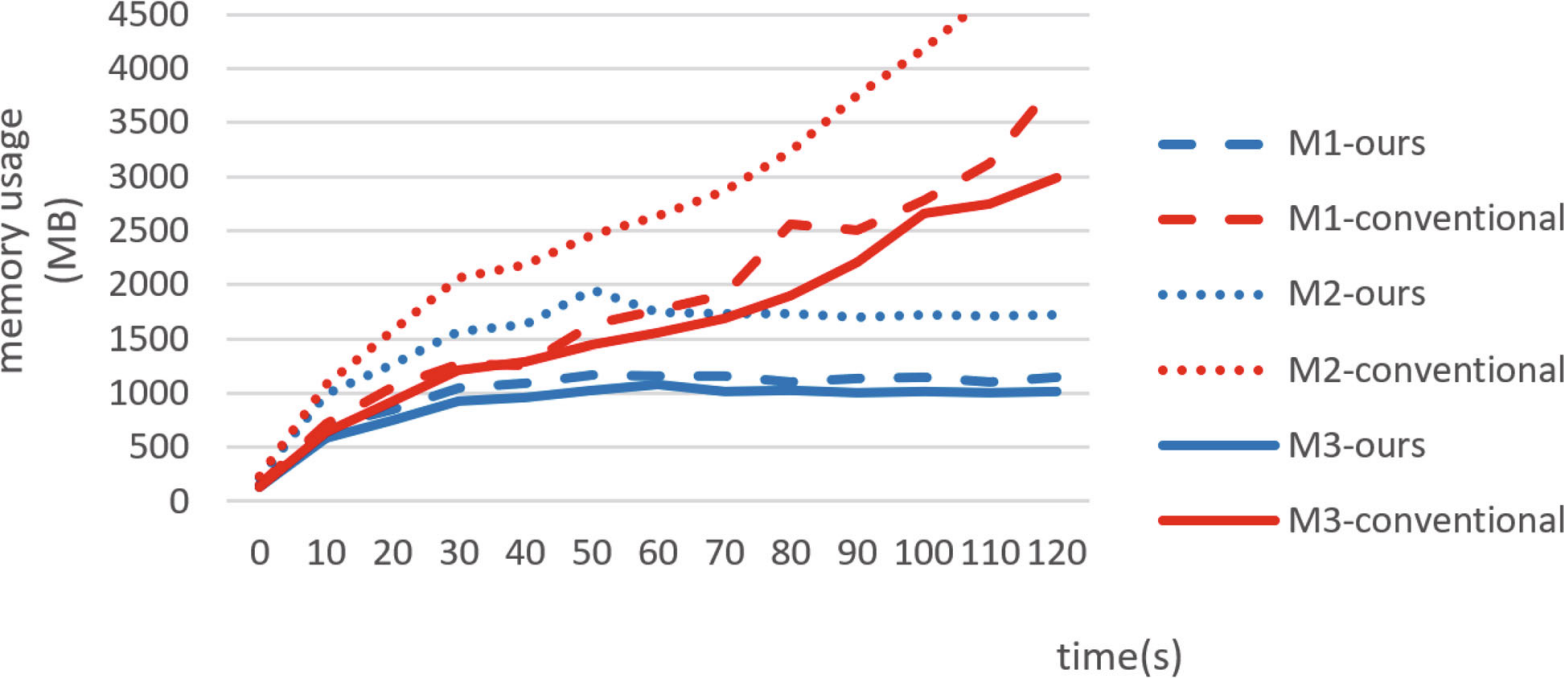
Memory usage of the test models

In order to analyze resource occupation, we monitor the memory footprint on the client. As shown in Fig. 8, it consumes over 4.5 GB memory by the conventional method that loads all IFC model data for displaying. Conversely, only 1.7 GB memory is consumed in the proposed method. This is mainly because the proposed method loads only part of the IFC model (i.e., external building components and specific story components) as opposed to loading the complete model. With respect to the three test models, the proposed method reduces the memory occupied by approximately 70%.

### Analysis of dynamically loading speed

**Fig. 9:**
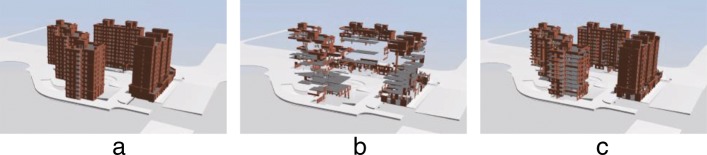
Comparison between the conventional dynamically loading method and proposed model for the same industry foundation classes file where 35% data of the same model is loaded for the two methods. **a** The whole model **b** Conventional method with 35% data loaded **c** Our method with 35% data loaded

In order to analyze the loading speed of the proposed method, we first compare the display effect of dynamically loading while using the conventional dynamically loading method and the proposed method for the same IFC file where the conventional method randomly dynamically loads IFC model data. Figure 9b and c show the display results where 35% data of the same model is loaded for the two methods. The results indicate that the proposed method presents a good overview of the model while only loading 35% of the data (or even less). Conversely, the randomly loading method only shows a small portion of external building components and does not display the overview of the model well.

In Table 2, we list the loading time of the test models. In the table, “Time1(s)” denotes the time of loading 35% data by the proposed method, “Time2(s)” denotes the time of loading 100% data by the proposed method, and “Time3(s)” denotes the time of loading 100% data by the conventional method. Specifically, the time for the proposed method to load the complete model is almost identical to that for the conventional method. Here, only 35% of the time (or even less) is required to obtain the overview of the model by the proposed method, and this significantly improves user experience.

**Table 2:** Loading times of the test models for the conventional method and proposed method

Model name	Time1(s)	Time2(s)	Time3(s)
M1	12	39	41
M2	20	54	49
M3	11	40	42

### Analysis of display effect

**Fig. 10:**
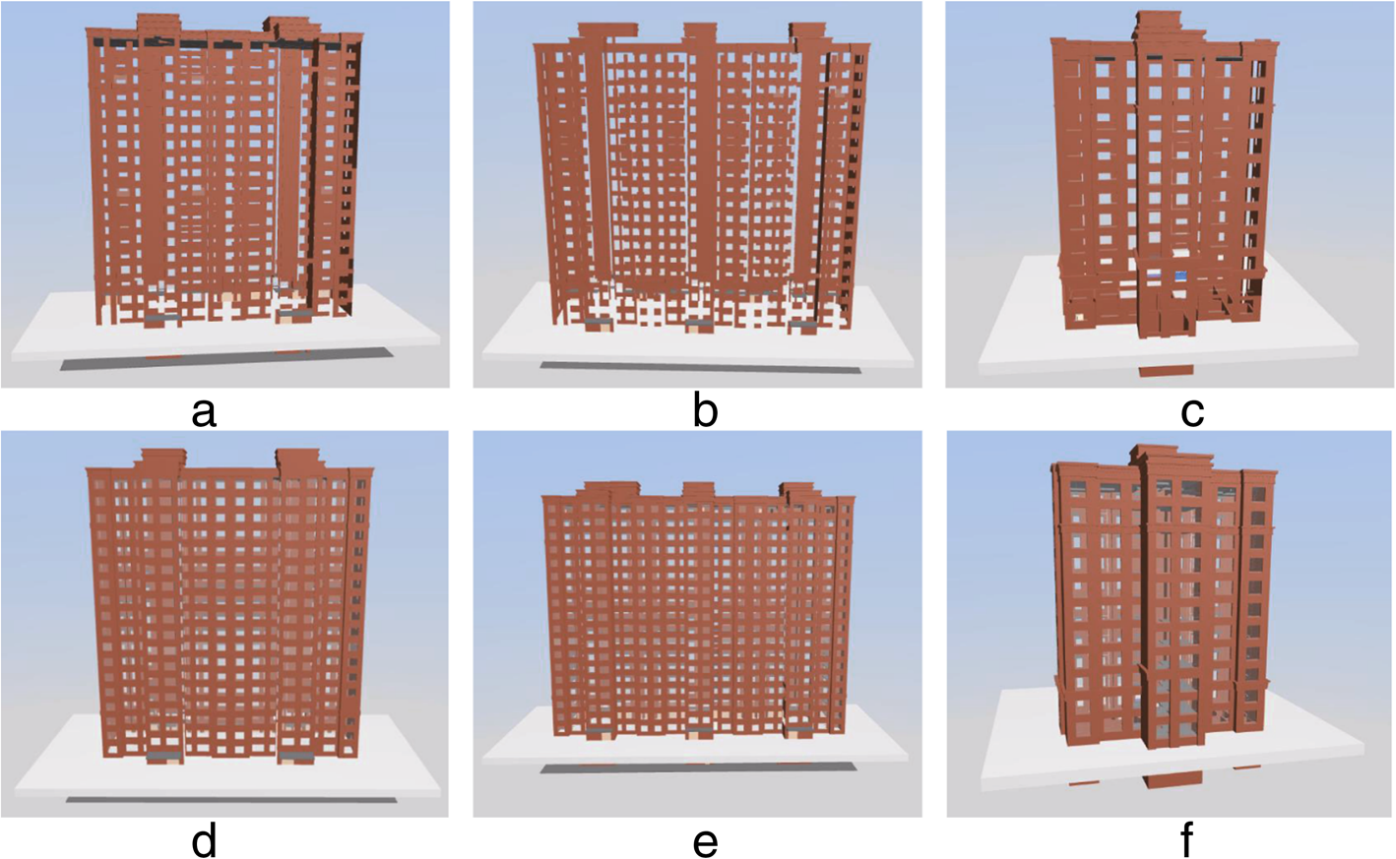
Comparison of the results of the external component extraction between the bounding box based method and proposed method. **a** M1 **b** M2 **c** M3 **d** M1 **e** M2 **f** M3

In order to analyze the display effect of external component extraction, three models are compared with the external component extraction algorithm based on the bounding box and the external component extraction algorithm according to node classification. The experimental results are shown in Fig. 10. Figure 10a, b and c denote the results of the extraction algorithm based on the bounding box. Figure 10d, e and f denote the results of the external component extraction via the proposed method.

As shown in Fig. 10, the external component extraction algorithm based on the bounding box misses several external windows and doors while extracting the external components of the three test models. The main reason is described as follows. The bounding box of walls contains the bounding box of windows and doors, and thus the windows and doors overlap in two-dimensional projection and lead to an omission. In the study, the algorithm is based on the intersection of each node and component of the node classification. When a component is included in another component, the algorithm continues to intersect with the node. Therefore, the proposed method accurately extracts the external components.

**Fig. 11:**
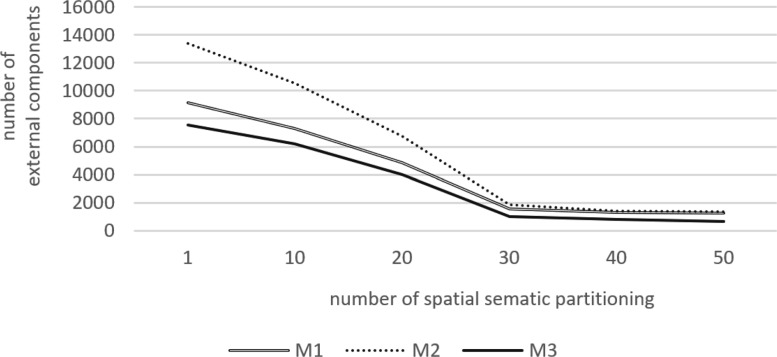
Results of extracting external components

Figure 11 shows the changes in the number of external components extracted from the three models based on the different partitions. As shown in Fig. 11, the number of extracted external components decreases when the number of partitions increases. When the number of partitions exceeds 30, the number of extracted external components tends to be stable because the model space is already divided into 27,000 cube nodes, and thus the components cover the nodes more uniformly. The aforementioned analysis indicates that the number of external components typically accounts for a small proportion of the total number of components. At the time of preloading, only the extracted external components are displayed, and this effectively reduces the amount of model data when compared to the display of the complete model data.

### Analysis of operation fluency

In order to evaluate the operational fluency, we use the frames per second (FPS). Under normal circumstances, the operation is fluent when the frame rate exceeds 30FPS. A user experiences a certain delay when the frame rate is lower than 20FPS. It’s unbearable when the frame rate is less than 10FPS. Therefore, in an ideal case, the frame rate should be maintained above 20FPS. Table 3 shows the FPS between the proposed method and the conventional method where “FPS1” denotes the FPS of the proposed method when only the external components are shown, “FPS2” denotes the FPS when only the internal components are shown, and “FPS3” denotes the FPS of the conventional method.

**Table 3:** FPS of the test models

Model name	FPS1	FPS2	FPS3
M1	35	39	4
M2	20	28	3
M3	40	46	5

The proposed method only displays part of the model, and this reduces the rendering pressure on the client. As shown in Table 3, the frame rate of M2 is only 3FPS when the method is not optimized. In the proposed method, the frame rate increases to 20FPS, and users can clearly view the model.

## Conclusion

The study proposes a method for dynamically loading IFC models based on SSP. Only the external components of the display model were loaded in the initial loading of the model. The model was divided by story information such that it could be dynamically loaded and displayed by interactive operation. Additionally, the geometric data was cached, and this avoided the repeated downloading of the same geometric data. We implemented the presented method with WebGL, and this enabled fast loading of large IFC models on the web browser without any plug-ins. The experimental results indicated that the proposed method significantly reduced the memory consumption in a web browser, and this allowed fast loading of large IFC models and provided a better interactive experience for users.

The current implementation of the proposed method still exhibited a few disadvantages. One of the limitations is that the algorithm of external component extraction based on node classification is not sufficiently robust for non-closed BIM models. For example, mechanical, electrical, and plumbing (MEP) designer models and structural designer models are typically provided individually, and this may not explicitly include the architecture models. Thus, it is difficult to distinguish between external components and internal ones while only using MEP and structural models. In the future, we will explore more effective algorithms of external component extraction and especially for MEP and structural models. Additionally, a more effective memory management strategy is also an important method to improve the performance of dynamically loading IFC models, and this will be explored in a future study.
